# Cerebral Autosomal Dominant Arteriopathy with Subcortical Infarcts and Leukoencephalopathy (CADASIL) as a model of small vessel disease: update on clinical, diagnostic, and management aspects

**DOI:** 10.1186/s12916-017-0778-8

**Published:** 2017-02-24

**Authors:** Ilaria Di Donato, Silvia Bianchi, Nicola De Stefano, Martin Dichgans, Maria Teresa Dotti, Marco Duering, Eric Jouvent, Amos D. Korczyn, Saskia A. J. Lesnik-Oberstein, Alessandro Malandrini, Hugh S. Markus, Leonardo Pantoni, Silvana Penco, Alessandra Rufa, Osman Sinanović, Dragan Stojanov, Antonio Federico

**Affiliations:** 1 Department of Medicine, Surgery and Neurosciences, Medical School, University of Siena, Viale Bracci 2, 53100 Siena, Italy; 2 Institute for Stroke and Dementia Research, Klinikum der Universität München, Ludwig-Maximilians-University LMU, Munich, Germany; 3 Munich Cluster for Systems Neurology (SyNergy), Munich, Germany; 4 Université Paris Diderot, Sorbonne Paris Cité, UMR-S 1161 INSERM, F-75205 Paris, France; 5 Department of Neurology, AP-HP, Lariboisière Hospital, F-75475 Paris, France; 6 DHU NeuroVasc Sorbonne Paris Cité, Paris, France; 7 Department of Neurology, Tel Aviv University, Ramat Aviv, 69978 Israel; 8 Department of Clinical Genetics, K5-R Leiden University Medical Center, PO Box 9600, 2300 RC Leiden, The Netherlands; 9 Stroke Research Group, Department of Clinical Neurosciences, University of Cambridge, Cambridge, UK; 10 NEUROFARBA Department, Neuroscience section, Largo Brambilla 3, 50134 Florence, Italy; 11 Medical Genetic Unit, Department of Laboratory Medicine, Niguarda Hospital, Milan, Italy; 12 Department of Neurology, University Clinical Center Tuzla, School of Medicine University of Tuzla, 75000 Tuzla, Bosnia and Herzegovina; 13 Faculty of Medicine, University of Nis, Bul. Dr. Zorana Djindjica 81, Nis, 18000 Serbia

**Keywords:** CADASIL, Small vessel disease, *NOTCH 3*, Vascular dementia, Genetics

## Abstract

Cerebral autosomal dominant arteriopathy with subcortical infarcts and leukoencephalopathy (CADASIL) is the most common and best known monogenic small vessel disease. Here, we review the clinical, neuroimaging, neuropathological, genetic, and therapeutic aspects based on the most relevant articles published between 1994 and 2016 and on the personal experience of the authors, all directly involved in CADASIL research and care. We conclude with some suggestions that may help in the clinical practice and management of these patients.

## Background

Cerebral small vessel disease (SVD) is an important cause of stroke, cognitive impairment, and mood disorders in the elderly. Besides the common sporadic forms, mostly related to age and hypertension, a minority of SVD has a monogenic cause, among which the most common and best known is cerebral autosomal dominant arteriopathy with subcortical infarcts and leukoencephalopathy (CADASIL). CADASIL provides a unique model for the study of the most prevalent forms of sporadic SVD. CADASIL is caused by mutations in the *NOTCH3* gene, which maps to the short arm of chromosome 19 and encodes the NOTCH3 receptor protein, predominantly expressed in adults by vascular smooth muscle cells and pericytes [1]. Thousands of families with CADASIL have now been diagnosed worldwide in many different ethnic groups. The disorder is often overlooked and misdiagnosed. Its minimum prevalence has been estimated between 2 and 5 in 100,000 but may vary between populations [2–5].

In this article, we discuss the main clinical, genetic, neuroimaging, neuropathological, and management aspects of the disease.

## Methods

**Fig. 1:**
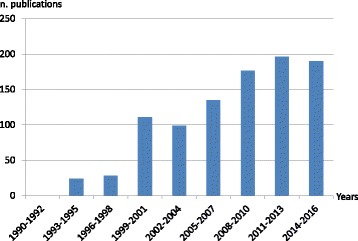
Results on PubMed search by years 1993–2016

The project for this paper was developed following the 9th International Congress on Vascular Dementia, 2015, held in Ljubljana, Slovenia. Articles published between 1994 to 2016 with the keyword ‘CADASIL’ were retrieved from the PubMed database. Figure 1 reports the number of PubMed articles on CADASIL during the past years. Only English language papers with high scientific relevance were reviewed. In total, 163 articles related to CADASIL were reviewed and included in the paper.

## Clinical aspects

The main clinical features include migraine usually with aura presenting in early adulthood [6], recurrent subcortical ischemic events, mood disturbances [7], progressive cognitive impairment mostly affecting executive function, and acute encephalopathy [8, 9]. Pescini et al*.* [10] developed a score which may be helpful in confirming the clinical suspicion of CADASIL and identifying patients in whom mutation screening has a reasonable yield.

### Migraine

Migraine is often the earliest feature of the disease, being reported in about 55–75% of Caucasian cases, although it is less frequent in Asian populations [11]. The age at onset is highly variable, generally around 30 years [12]. In a recent article on the prevalence and characteristics of migraine in 378 CADASIL patients [6], a total of 54.5% of individuals had a history of migraine and 84% of these had migraine with aura (MA). Women with MA accounted for 62.4% of the total, with an earlier MA onset compared to men. Atypical auras, such as prolonged visual auras, gastrointestinal manifestations, dysarthria, confusion, focal neurologic deficits, and other uncommon manifestations, were experienced by 59.3% of individuals with MA, and in 19.7% of patients the aura was sine migraine. MA was the first clinical manifestation in 41% of symptomatic patients and an isolated symptom in 12.1%. The pathophysiological reasons leading to increased aura prevalence in CADASIL are unknown; a possible mechanism is an increased susceptibility to cortical spreading depression [13]. It is also possible that migraine in CADASIL involves the brainstem region. Alternatively, the *NOTCH3* mutation itself may act as a MA susceptibility gene [14].

### Subcortical ischemic events

Transient ischemic attacks and stroke are reported in approximately 85% of symptomatic individuals [15] and are related to cerebral small vessel pathology. Mean age at onset of ischemic episodes is approximately 45–50 years [16], but the range at onset is broad (3rd to 8th decade).

Ischemic episodes typically present as a classical lacunar syndrome (pure motor stroke, ataxic hemiparesis/dysarthria-clumsy hand syndrome, pure sensory stroke, sensorimotor stroke), but other lacunar syndromes (brain stem or hemispheric) are also observed. The total lacunar lesion load, symptomatic and asymptomatic, is strongly associated with the development of severe disability with gait disturbance, urinary incontinence, pseudobulbar palsy and cognitive impairment. Strokes involving the territory of a large artery have occasionally been reported but whether these are co-incidental or related to the CADASIL pathology itself is uncertain [17–19]. There have been a few cases of subcortical hemorrhage, mostly in patients taking anticoagulants.

### Encephalopathy

An acute encephalopathy [20] has been described in 10% of CADASIL patients, and in the majority of these it was the first major symptom, with a mean age of onset of 42 years [9]. It is frequently misdiagnosed as encephalitis, particularly if it is the initial presentation in a patient without known CADASIL. It usually evolves from a migraine attack, including confusion, reduced consciousness, seizures, and cortical signs, with spontaneous resolution.

Cognitive impairment in CADASIL first involves information processing speed and executive functions, with relative preservation of episodic memory, and is associated with apathy and depression [21–25]. Memory impairment has been reported, particularly later in the disease. Cognitive screening measures, as the Brief Memory and Executive Test and the Montreal Cognitive Assessment, are clinically useful and sensitive screening measures for early cognitive impairment in patients with CADASIL [26]; 3-year changes in Mini Mental State Examination, Mattis Dementia Rating Scale, Trail Making Test version B, and modified Rankin Scale are validated models for prediction of clinical course in CADASIL [27]. The cognitive deficits were initially attributed to subcortical origin [28]. More recently, the cerebral cortex was shown also to be affected in CADASIL [29] by direct mechanisms (cortical microinfarcts [30]), or through secondary degeneration [31]. Memory impairment in CADASIL is probably related to different causes, due to both white matter infarcts with disruption of either cortico-cortical or subcortical-cortical networks mainly in the frontal lobe and primary damage of the cortex [32, 33]. In a recent longitudinal study, processing speed slowing was related to a decrease of sulcal depth, but not to global brain atrophy or cortical thinning, suggesting that early cognitive changes may be more specifically related to sulcal morphology than to other anatomical changes [34]. Moreover, studies in mouse models of CADASIL have detected dysregulation of hippocampal neurogenesis, a process essential for the integration of new spatial memory records [35].

### Psychiatric disturbances

Although the clinical expression of the disease is mainly neurological, CADASIL is also characterized by psychiatric disturbances (20–41%) [36, 37]. Apathy and major depression are commonly observed in CADASIL. Also bipolar disorder and emotional incontinence are present in a consistent percentage of patients [38]. The direct consequence is a negative effect on patient’s quality of life and caregiver burden, with different degrees. Other psychiatric manifestations, such as psychotic disorders, adjustment disorders, personality disorders, drug addiction, and abuse of substances, are less frequent. The pathogenesis of psychiatric disturbances in CADASIL is incompletely understood, but, similarly to other cerebrovascular diseases, may depend on the damage of the cortical–subcortical circuits, leading to the consideration of mood disorders in CADASIL under the concept of ‘vascular depression’.

In conclusion, CADASIL symptoms may be highly variable, usually starting in adulthood, but also reported in older age [39]. The wide clinical spectrum includes complex migraine attacks with prominent aura, acute confusional states or coma, lacunar strokes, and pure psychiatric or cognitive presentations. Ischemic events, in different brain areas, may involve also the optic nerve [40]; retinal involvement is also described [41, 42]. Few reports also suggest the presence of myopathy or peripheral neuropathy, the first possibly related to involvement of mitochondrial dysfunctions, but these findings may be coincidental. Typical and atypical phenotypes are reported in Table 1.

**Table 1:** Typical and atypical clinical manifestations of CADASIL

Typical manifestations	
Migraine, usually with aura, as the first symptom in the third decade of life	
Recurrent subcortical ischemic events (transient ischemic attack/stroke) in adulthood	
Mood disturbances, apathy and depression among other psychiatric symptoms	
Progressive cognitive decline, especially of executive functioning	
Seizures, in a smaller but well-defined portion of patients	
Atypical manifestations	
Pathological gambling [128]	
Recurrent status epilecticus [129]	
Schizopheniform organic psychosis [130, 131]	
Neuropathy [132–134]	
Myopathy [100, 135–138]	
‘CADASIL coma’ [25, 139]	
Early onset [140, 141]	
Late onset [39]	
Bipolar disorder [142]	
Inflammatory-like presentation [143]	
Acute vestibular syndrome [144]	
Spinal cord involvement [145, 146]	
Acute confusional migraine [147]	
Sporadic hemiplegic migraine with normal imaging [148]	
Post-partum psychiatric disturbances [149]	
Parkinsonism [150]	
Recurrent transient global amnesia [151]	

## Modulating factors

CADASIL is characterized by a large phenotypic variability both across and within families. No clear genotype–phenotype correlation has been found to date [43] and both modulating genetic and risk/environmental factors probably play a role.

Among the conventional risk factors, hypertension and smoking are associated with early age of onset of stroke [9, 43, 44] and with clinical worsening towards dementia [45, 46]. In a recent longitudinal study, smoking was associated with a three-fold increase in stroke risk [46]. Homocysteine levels have been found to be higher in patients with CADASIL who have migraine, but this finding needs replicating [43].

Opherk et al. [47, 48] found that white matter lesion volumes are influenced by genetic factors distinct from the causative *NOTCH3* mutation, but the nature of the responsible modulating genetic factors is currently unknown. The role of Apolipoprotein E (APOE) is debated – Gesierich et al. [49] suggested a modifying influence of APOE ɛ2 on the volume of white matter hyperintensities, while Singhal et al. [43] detected no relationship between phenotype and APOE ɛ4 genotype.

Gunda et al*.* [50] hypothesized on a possible role of ovarian hormones in sex-related differences in CADASIL, data that need further confirmation.

Recently, Pescini et al. [51] evaluated a possible role of plasma levels of von Willebrand factor, blood levels of endothelial progenitor cells, and endothelial impairment in the pathogenesis of the disease.

## Life expectancy, cause of death, and cardiovascular aspects

Life expectancy is reduced in CADASIL patients. An age at death in men of 64.6 years and in women of 70.7 years has been reported in a large study of 411 subjects [16]. Pneumonia in patients with disability was the major cause of death (38%), but a high number of sudden unexpected deaths were also observed, accounting for up to 26% [16]. Whether cardiac arrhythmias, QT variability index, and myocardial infarction are more frequent in CADASIL than in the general population remains to be confirmed [52–55].

Another point of interest is the finding of a non-dipper pattern of nocturnal blood pressure [56]. A lower nocturnal blood pressure fall may be associated with incidence and/or worsening of deep white matter lesions in CADASIL. The pathogenesis of abnormal blood pressure profile in CADASIL remains to be clarified. Central and peripheral mechanisms controlling blood pressure variations may be involved.

The association between CADASIL and right-to-left cardiac shunt has been reported but remains under debate [57, 58].

## Neuroimaging

**Fig. 2:**
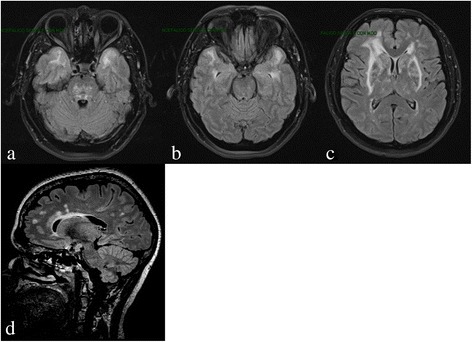
Axial FLAIR MRI: Multifocal/confluent subcortical white matter lesions, with involvement of anterior temporal lobes (**a**, **b**), pons (**a**), external capsules, periventricular and fronto-pariental regions (**c**), and corpus callosum (**d**)

Cerebral Magnetic Resonance Imaging (MRI) in CADASIL shows age- and disease stage-dependent extensive white matter abnormalities, seen as large symmetrical hyperintense signals on T2-weighted and FLAIR images [59], and lacunes, i.e., fluid-filled cavities with cerebrospinal fluid-isointense signal. Diffusion-weighted imaging may show acute and subacute infarctions, and can also be used to quantify chronic white matter changes through diffusion tensor metrics. White matter changes often involve the anterior temporal lobe, the external capsule and the superior frontal gyrus [60]. Anterior temporal pole changes have been shown to have a high sensitivity and specificity for the disease (approximatively 90% for each) and are useful in diagnosis [61]. In Asian populations, anterior temporal lobe involvement is less common [62]. External capsule changes also have a high sensitivity (approximatively 90%) but a lower specificity (approximately 50%). In fact, a recent systematic analysis showed a similar involvement of the external capsule in CADASIL and sporadic SVD [63]. Corpus callosal signal abnormalities, rarely occurring in sporadic SVD, are described in CADASIL [59] (Fig. 2); such abnormalities are also a feature of multiple sclerosis, which is one reason for the misdiagnosis of CADASIL as multiple sclerosis. Cerebral microbleeds, which are shown by Gradient-echo images with dot-like hypointense lesions, occur in a variable proportion of cases (30–70%) and usually increase with age and risk factors such as high blood pressure, and intracerebral hemorrhage have also been described in a number of patients [64–66], especially those of Asian origin [67]. Nevertheless, the clinical significance of the cerebral microbleeds observed in CADASIL has not been clearly elucidated.

MRI white matter signal abnormalities are found in presymptomatic CADASIL mutation carriers aged 20–30 years or older [59, 68].

The MRI findings that have been most strongly related to clinical deficit are number and volume of lacunes, brain atrophy, and white matter damage as assessed by diffusion tensor imaging [69, 70]. CADASIL has primarily been defined as a subcortical disease, but recent studies with high-field 7-Tesla MRI, which allows higher resolution imaging, have detected primary involvement of the cortex, including the demonstration of cortical microinfarcts and early diffuse cortical alterations [23] in frontal and parietal regions most often following a symmetrical pattern in both hemispheres. These changes were not related to cortical thinning or to subcortical lesions and have been hypothesized to be secondary to venous vascular density or intramyelin edema [71]. Similar data have also been reported in experimental models [72]. Moreover, subcortical changes might induce secondary cortical changes [69, 72–74]. In a longitudinal study, incident lacunes were followed by cortical thinning specifically in connected brain regions [31]. Another recent development is the use of diffusion tensor imaging to characterize tissue damage [75, 76]. The typical change in diffusion metrics observed in CADASIL patients is a reduction in fractional anisotropy (a measure for the directionality of diffusion) and an increase in the apparent diffusion coefficient or mean diffusivity (a measure for the extent of diffusion) [69, 77]. Histogram analysis has been proposed as a sensitive tool to measure CADASIL-related changes cross-sectionally as well as longitudinally [78–80].

## Neuropathology

The pathological hallmark of CADASIL is deposition of granular osmiophilic material (GOM) in close relation to vascular smooth muscle cells, seen on electron microscopy. GOM can also be detected in vessels of extracerebral tissues, including skin and muscle. Consequently, skin biopsy has been considered an important diagnostic tool [81], with a specificity of 100%, and variable sensitivity from 45% to 100% according to the different reports [82–84]. The reasons for this discrepancy may be related to different factors, including different stages of the disorder, the size of the biopsy and the number of arteriolar and precapillary vessels in the specimen, or the focality of lesions. It is also important to differentiate GOM from dense bodies and non-specific granular debris present in degenerated smooth muscle cells. The reason why the small-to-medium sized cerebral arteries are most severely affected and why there are no extra-cerebral clinical disease manifestations is still not fully understood. Further, the composition of GOM remains partly unclear. Previous immunoelectron microscopy studies reported the accumulation of the NOTCH3 ectodomain in proximity of GOM formation [85]. More recent investigation by post-embedding immunoelectron microscopy analysis using antibodies against the extra- and intracellular portions of *NOTCH3*, showed the *NOTCH3* ectodomain to be a major component of GOM [86–88]. Immunohistochemistry analysis using a NOTCH3 monoclonal antibody was proposed as a further reliable tool for CADASIL diagnosis [89]. Although high specificity and sensitivity, ranging from 90% to 100%, were reported, this high level of accuracy has not been reported by any other group and the technique is not currently widely used in diagnosis [90].

In conclusion, preferably, skin biopsy analysis should include both *NOTCH3* immunohistochemistry and electron microscopy. For electron microscopy analysis at least 4–6 arteriolar vessels for each patient should be examined. In most CADASIL centers, skin biopsy is only reserved in those cases with suspected CADASIL, but who did not have a *NOTCH3* mutation or who had an unclassified variant in *NOTCH3*. Genetic testing is becoming an increasingly accessible diagnostic tool and, in patients with a clinical suspicion of CADASIL, *NOTCH3* Sanger sequencing of EGFr encoding exons, if available, is the first choice for confirming the clinical diagnosis. This has the added advantage that testing of family members (both symptomatic and asymptomatic) is feasible once the mutation is known in the index patient.

There are few brain neuropathological analyses published in CADASIL, and these have mainly focused on cerebral vessel changes that are reported to be consistently narrowed with intimal thickening and degeneration of vascular smooth muscle cells and GOM deposition [91]. In 11 patients, Craggs et al*.* [92] reported disruption of either cortico-cortical or subcortico-cortical networks in the white matter of the frontal lobe, explaining motor deficits and executive dysfunction present in CADASIL.

## Genetics

*NOTCH3* is the causative gene for CADASIL, which maps to the short arm of chromosome 19 (19p13.2-p13.1) [1, 93]. It is a large gene containing 33 exons [94]. The *NOTCH3* gene codes for a single-pass transmembrane receptor protein, with a large extracellular domain, with 34 EGFRs (tandem epidermal growth factor-like repeat domains) and 3 LNR repeats, a transmembrane portion and an intracellular domain containing ankyrin-repeat motifs and a PEST domain [95]. NOTCH3 protein is expressed in vascular smooth muscle cells, whose degeneration results in progressively impaired cerebrovascular autoregulation, hypoperfusion, and ischemia [84]*.* More than 200 different *NOTCH3* gene CADASIL-associated mutations have been reported, the majority of them consisting of stereotyped missense substitutions of a single base leading to loss or gain of a cysteine residue within one of the EGFRs of *NOTCH3* extracellular domain [96]. Initially, most mutations were found to cluster in exons 3 and 4, but there are now many reports of mutations in other exons encoding the extracellular portion of the protein [2, 39, 97, 98]. Therefore, screening should include all EGFR encoding exons (exon 2–24). A few patients are reported with homozygous or compound heterozygous (Dotti unpublished data, [99]) *NOTCH3* mutations; in these patients, the phenotype is almost always within the classical CADASIL spectrum [100–105].

Whether non-cysteine variants can cause CADASIL is debated. Small *NOTCH3* deletions, duplications, splice site mutations, and deletion/insertions associated with CADASIL have been described. Most of these mutations lead to a numerical cysteine acid change [106]. In one study, a small intronic deletion leading to intron 3 retention was found to be associated with a GOM-positive CADASIL patient; the 25 additional amino acids in the EGFR likely disturb the disulphide bond formation [107]. Several studies have reported atypical, cysteine-sparing, *NOTCH3* mutations associated with a familial CADASIL phenotype. These variants may be interpreted as causative mutations only if the complete screening of the EGFR-encoding exons excludes other classic mutations and GOM are present in skin biopsy [106, 108, 109]. However, one study suggested that GOM analysis might be less sensitive in patients with non-cysteine involving mutations [110].

Mutations causing loss of function (frame-shift deletion or nonsense mutations) are rarely reported in CADASIL patients, and their role is still debated [106, 111, 112]; they should be carefully analyzed to determine their pathogenicity. *NOTCH3* mutations located outside of the EGFR encoding exons, reported in few cases, are not associated with a CADASIL phenotype [113–115].

**Fig. 3:**
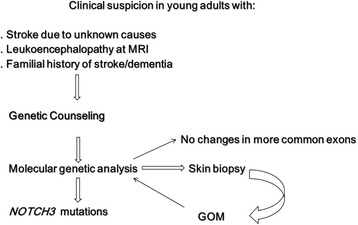
Flow-chart for CADASIL diagnosis

A simple flow chart for CADASIL diagnosis is shown in Fig. 3.

## Genetic counseling

Genetic counseling should be offered to all patients diagnosed with CADASIL and should always precede predictive genetic testing of family members. For predictive genetic testing, the Huntington’s disease guidelines can be used [116, 117]. It is important to note that a MRI scan in an unaffected family member can have a similar impact as a genetic test if it shows the characteristic MRI hallmarks of the disease. Genetic counselling also plays an important role in family planning and questions about options for prenatal diagnosis or pre-implantation genetic diagnosis may be discussed in countries where these techniques are available [118, 119].

## Treatment

An effective treatment for CADASIL is not available. In the absence of curative approaches, treatments should be directed towards the search of possible disease-modifying strategies to mitigate clinical manifestations [120]. The review of available data shows that only a few and very preliminary studies have been reported thus far. In Tables 2 and 3, we summarized studies focused on symptomatic outcomes and those focused on cerebral and peripheral blood flow changes with a possible effect on disease mechanisms.

**Table 2:** Symptomatic treatment in CADASIL

Symptom	Therapy	Effects
Migraine	Acetazolamide [152–154]	As prophylaxis, reduces the frequency of migraine attacks
	Sodium valproate [155]	Anecdotal cases
Cognitive decline	Acetylcholinesterase inhibitor [121]	Not efficacious on the primary end-point (Vascular Dementia Assessment scale after 18 weeks), but some improvement in relation to frontal-subcortical dysfunction
Primary and secondary stroke prevention	Antiplatelet drugs [66, 123]	Unproven and debated benefits

**Table 3:** Therapeutic studies on blood flow in CADASIL

Drug	Effect
Acetazolamide	Related to an increase of cerebral perfusion evaluated by perfusion MRI [156], transcranial Doppler sonography [157] and Tc-99 m extracellular domain brain perfusion SPECT [158, 159]
Atorvastatin	No effects on cerebral blood flow [160], tested with transcranial Doppler
L-arginine	Induced-vasoreactivity [161], tested with transcranial Doppler
Sapropterin (200–400 mg bid in 24 months)	Final results for the primary end-point (mean difference in reactive hyperemia index) were not significant for any improvement of peripheral vasoreactivity [162]
Novel molecular therapeutic target (as stem cell factor and granulocyte-colony stimulating factor)	Ongoing [163]

In view of the evidence of a more severe disease course in individuals with vascular risk factors, particularly smoking and hypertension, control of vascular risk factors is an important part of CADASIL management. Concerning the use of antiplatelet drugs, such as aspirin or clopidogrel, most neurologists apply the guidelines used for sporadic stroke when treating CADASIL patients. However, the appropriateness of this approach is undetermined. In fact, the thrombotic genesis of ischemic events in this disease has not been proven so far. On the other hand, many reports have stressed the presence of microhemorrhages (microbleeds) in a considerable percentage of CADASIL patients [121]. For these reasons, the safety of antiplatelet drugs in this disease remain to be clarified [65]. Similarly, the benefit of thrombolysis is uncertain, although it has been suggested that it is of benefit in sporadic lacunar stroke [122].

Following the data reported by Keverne et al*.* [121] showing a cholinergic neuronal impairment in CADASIL, a multicenter trial in 168 patients with donepezil was performed; this showed no improvement in the primary outcome of the Vascular Dementia Assessment scale – cognitive subscale. Improvements were noted on several measures of executive function, but the clinical relevance of these findings is not clear [123]. Complications of CADASIL, such as depression and migraine, appear to respond to similar treatments to those used in sporadic disease, although further studies are required to determine the efficacy of these approaches in CADASIL patients.

## Differential diagnosis

A differential diagnosis in some cases may be hard. However, in CADASIL, in comparison to multiple sclerosis, temporo-polar MRI lesions are present in a high percentage, the optic nerve and spinal cord are generally not involved, and oligoclonal bands in the cerebrospinal fluid are absent. Corpus callosum hyperintensities are described both in CADASIL and in multiple sclerosis [124, 125]. In relation to cerebral autosomal dominant arteriopathy with subcortical infarcts and leukoencephalopathy (CARASIL), in CADASIL, extraneurological findings (early-onset spondylosis and early-onset alopecia) are absent, clinical onset is generally later, MA is a typical sign, and inheritance is autosomal dominant [126]. Other hereditary leukoencephalopathies to take into account for a correct differential diagnosis are MELAS, Krabbe disease, Fabry disease, CARASAL, hereditary diffuse leukoencephalopathy with axonal spheroids, and other more rare monogenic forms of SVD [127].

### Highlights and key questions for management of CADASIL patients

- **Clinical presentation:** The clinical presentation is usually stereotyped with MA, small subcortical strokes, or cognitive impairment in middle-aged patients with a family history of stroke or dementia, but the spectrum of the disease is wide and atypical phenotypes have been reported. There is wide variation in phenotype both within and between families.
- **Cognitive impairment:** Cognitive impairment is a prominent feature in CADASIL. While its clinical pattern is typically subcortical, several recent data suggest that the cerebral cortex is also involved and contributes to the cognitive profile abnormalities.
- **Brain MRI:** Cerebral MRI in CADASIL shows extensive white matter changes, with frequent involvement of temporal pole and external capsule. No abnormality is pathognomonic, but confluent bilateral anterior temporal pole T2-hyperintensities are highly suggestive. Recent advances in neuroimaging have also revealed involvement of the cortex.
- **Vascular risk factors:** Smoking seems to be clearly associated with an increase in the stroke risk both in cross-sectional and longitudinal studies. Moreover, smoking and hypertension are associated with an earlier onset of stroke and more rapid progression in CADASIL. Evidence is less robust for other risk factors and more study is needed. However, in the absence of effective therapeutic approaches, control of vascular risk factors is recommended. Also, genetic modulating factors other than the *NOTCH3* mutation probably play a role, but their nature remains to be determined.
- **Genetic:** More than 200 different *NOTCH3* gene mutations have been reported in exons 2–24, involving the loss or gain of cysteine residues. The role of non-canonic *NOTCH3* mutations (e.g., cysteine-sparing mutations, mutations outside EGFR domains, frameshift or nonsense mutations) is debated; in these cases, the search for GOMs in skin is mandatory, although in some cases it may be negative. When identifying a non-canonic mutation, sequencing of all EGFR-encoding exons of the *NOTCH3* gene is mandatory as well as the cosegregation analysis of the non-canonic mutation with the CADASIL MRI phenotype within the family.
- **Neuropathology:** GOM is the neuropathological hallmark of CADASIL. Specificity is high, but sensitivity is variable. Mutation testing is the gold standard, and the use of skin biopsy should be limited to the rare situations when the diagnosis is strongly suspected despite negative genetic testing.
- **Genetic counseling:** Genetic counseling should always be offered to patients diagnosed with CADASIL and should precede all predictive genetic testing. Predictive genetic testing should only be offered to adults (>18 years) and should be performed by trained staff with genetic counselling experience, using a standard protocol such as that used in Huntington’s disease.
- **Treatment:** In the absence of specific data for CADASIL, most neurologists use aspirin in secondary prevention after ischemic strokes in older patients, for example, those aged over 40, but there is no evidence for or against its use. Whether this strategy is appropriate in CADASIL is undetermined and will require further investigation, given the possible increased hemorrhagic risk. Patients that need to undergo anticoagulation for a clear indication such as high risk atrial fibrillation should be carefully followed given the reported risk of intracerebral hemorrhage. A multicenter randomised controlled trial using donepezil to improve cognitive dysfunction showed no benefit on the primary endpoint and only improvements of uncertain clinical significance on several measures of executive function. New, rational therapeutic interventions for CADASIL are in early phases of pre-clinical development.

## Conclusions

CADASIL is the most frequent monogenic cerebral SVD, and has received a lot of attention in recent years as a model for SVD, with an increased number of cases. However, more work remains to be done in order to better explore the underlying mechanism of its pathogenesis. The studies on animal models (see elsewhere in this issue) will improve the understanding of the pathogenic mechanisms and may open new therapeutic strategies.

## References

[1] Joutel A, Corpechot C, Ducros A, Vahedi K, Chabriat H, Mouton P (1996). Notch3 mutations in CADASIL, a hereditary adult-onset condition causing stroke and dementia. Nature..

[2] Razvi SS, Davidson R, Bone I, Muir KW (2005). The prevalence of cerebral autosomal dominant arteriopathy with subcortical infarcts and leukoencephalopathy (CADASIL) in the west of Scotland. J Neurol Neurosurg Psychiatry.

[3] Narayan SK, Gorman G, Kalaria RN, Ford GA, Chinnery PF (2012). The minimum prevalence of CADASIL in northeast England. Neurology.

[4] Moreton FC, Razvi SS, Davidson R, Muir KW (2014). Changing clinical patterns and increasing prevalence in CADASIL. Acta Neurol Scand.

[5] Bianchi S, Zicari E, Carluccio A, Di Donato I, Pescini F, Nannucci S (2015). CADASIL in central Italy: a retrospective clinical and genetic study in 229 patients. J Neurol.

[6] Guey S, Mawet J, Hervé D, Duering M, Godin O, Jouvent E (2016). Prevalence and characteristics of migraine in CADASIL. Cephalalgia.

[7] Desmond DW, Moroney JT, Lynch T, Chan S, Chin SS, Mohr JP (1999). The natural history of CADASIL: a pooled analysis of previously published cases. Stroke.

[8] Chabriat H, Joutel A, Dichgans M, Tournier-Lasserve E, Bousser MG (2009). Cadasil. Lancet Neurol.

[9] Adib-Samii P, Brice G, Martin RJ, Markus HS (2010). Clinical spectrum of CADASIL and the effect of cardiovascular risk factors on phenotype: study in 200 consecutively recruited individuals. Stroke.

[10] Pescini F, Nannucci S, Bartaccini B, Salvadori E, Bianchi S, Ragno M (2012). The Cerebral Autosomal-Dominant Arteriopathy With Subcortical Infarcts and Leukoencephalopathy (CADASIL) Scale: a screening tool to select patients for NOTCH3 gene analysis. Stroke.

[11] Kim Y, Choi EJ, Choi CG, Kim G, Choi JH, Yoo HW (2006). Characteristics of CADASIL in Korea: a novel cysteine-sparing Notch3 mutation. Neurology.

[12] Tan RY, Markus H (2016). CADASIL: migraine, encephalopathy, stroke and their inter-relationships. PLoS One.

[13] Eikermann-Haerter K, Yuzawa I, Dilekoz E, Joutel A, Moskowitz MA, Ayata C (2011). Cerebral autosomal dominant arteriopathy with subcortical infarcts and leukoencephalopathy syndrome mutations increase susceptibility to spreading depression. Ann Neurol.

[14] Liem MK, Oberstein SA, van der Grond J, Ferrari MD, Haan J (2010). CADASIL and migraine: a narrative review. Cephalalgia.

[15] Dichgans M, Mayer M, Uttner I, Brűning R, Műller-Hőcker J, Rungger G (1998). The phenotypic spectrum of CADASIL: clinical findings in 102 cases. Ann Neurol..

[16] Opherk C, Peters N, Herzog J, Luedtke R, Dichgans M (2004). Long-term prognosis and causes of death in CADASIL: a retrospective study in 411 patients. Brain.

[17] Choi JC, Lee KH, Song SK, Lee JS, Kang SY, Kang JH (2013). Screening for NOTCH3 gene mutations among 151 consecutive Korean patients with acute ischemic stroke. J Stroke Cerebrovasc Dis.

[18] Yin X, Wu D, Wan J, Yan S, Lou M, Zhao G (2015). Cerebral autosomal dominant arteriopathy with subcortical infarcts and leukoencephalopathy: Phenotypic and mutational spectrum in patients from mainland China. Int J Neurosci.

[19] Kang HG, Kim JS (2015). Intracranial arterial disease in CADASIL patients. J Neurol Sci.

[20] Schon F, Martin RJ, Prevett M, Clough C, Enevoldson TP, Markus HS (2003). “CADASIL coma”: an underdiagnosed acute encephalopathy. J Neurol Neurosurg Psychiat..

[21] Peters N, Opherk C, Danek A, Ballard C, Herzog J, Dichgans M (2005). The pattern of cognitive performance in CADASIL: a monogenic condition leading to subcortical ischemic vascular dementia. Am J Psychiatry.

[22] Charlton RA, Morris RG, Nitkunan A, Markus HS (2006). The cognitive profiles of CADASIL and sporadic small vessel disease. Neurology.

[23] De Guio F, Reyes S, Vignaud A, Duering M, Ropele S, Duchesnay E (2014). In vivo high-resolution 7 Tesla MRI shows early and diffuse cortical alterations in CADASIL. PLoS One.

[24] Jouvent E, Reyes S, De Guio F, Chabriat H (2015). Reaction time is a marker of early cognitive and behavioural alterations in pure cerebral small vessel disease. J Alzheimer Dis.

[25] Reyes S, Viswanathan A, Godin O, Dufouil C, Benisty S, Hernandez K (2009). Apathy: a major symptom in CADASIL. Neurology.

[26] Brookes RL, Hollocks MJ, Tan RY, Morris RG, Markus HS (2016). Brief screening of vascular cognitive impairment in patients with cerebral autosomal-dominant arteriopathy with subcortical infarcts and leukoencephalopathy without dementia. Stroke.

[27] Jouvent E, Duchesnay E, Hadj-Selem F, De Guio F, Mangin JF, Hervé D (2016). Prediction of 3-year clinical course in CADASIL. Neurology.

[28] Romàn GC, Erkinjuntti T, Wallin A, Pantoni L, Chui HC (2002). Subcortical ischaemic vascular dementia. Lancet Neurol.

[29] Righart R, Duering M, Gonik M, Jouvent E, Reyes S, Hervé D (2013). Impact of regional cortical and subcortical changes on processing speed in cerebral small vessel disease. Neuroimage Clin..

[30] Jouvent E, Poupon C, Gray F, Paquet C, Mangin JF, Le Bihan D (2011). Intracortical infarcts in small vessel disease: a combined 7-T postmortem MRI and neuropathological case study in cerebral autosomal-dominant arteriopathy with subcortical infarcts and leukoencephalopathy. Stroke.

[31] Duering M, Righart R, Csanadi E, Jouvent E, Hervè D, Chabriat H (2012). Incident subcortical infarcts induce focal thinning in connected cortical regions. Neurology.

[32] Taillia H, Chabriat H, Kurtz A, Verin M, Levy C, Vahedi K (1998). Cognitive alterations in non-demented CADASIL patients. Cerebrovasc Dis.

[33] Amberla K, Wäljas M, Tuominen S, Almkvist O, Pőyhőnen M, Tuisku S (2004). Insidious cognitive decline in CADASIL. Stroke.

[34] Jouvent E, Mangin JF, Duchesnay E, Porcher R, Düring M, Mewald Y (2012). Longitudinal changes of cortical morphology in CADASIL. Neurobiol Aging.

[35] Ehret F, Vogler S, Pojar S, Elliott DA, Bradke F, Steiner B (2015). Mouse model of CADASIL reveals novel insights into Notch3 function in adult hippocampal neurogenesis. Neurobiol Dis..

[36] Valenti R, Poggesi A, Pescini F, Inzitari D, Pantoni L (2008). Psychiatric disturbances in CADASIL: a brief review. Acta Neurol Scand.

[37] Valenti R, Pescini F, Antonini S, Castellini G, Poggesi A, Bianchi S, Inzitari D, Pallanti S, Pantoni L (2011). Major depression and bipolar disorders in CADASIL: a study using the DSM-IV semi-structured interview. Acta Neurol Scand.

[38] Noh SM, Chung SJ, Kim KK, Kang DW, Lim YM, Kwon SU (2014). Emotional disturbance in CADASIL: its impact on quality of life and caregiver burden. Cerebrovasc Dis.

[39] Pescini F, Bianchi S, Salvadori E, Poggesi A, Dotti MT, Federico A (2008). A pathogenic mutation on exon 21 of the NOTCH3 gene causing CADASIL in an octogenarian paucisymptomatic patient. J Neurol Sci.

[40] Rufa A, De Stefano N, Dotti MT, Bianchi S, Sicurelli F, Stromillo ML (2004). Acute unilateral visual loss as the first symptom of cerebral autosomal dominant arteriopathy with subcortical infarcts and leukoencephalopathy. Arch Neurol.

[41] Rufa A, Pretegiani E, Frezzotti P, De Stefano N, Cevenini G, Dotti MT (2011). Retinal nerve fiber layer thinning in CADASIL: an optical coherence tomography and MRI study. Cerebrovasc Dis.

[42] Pretegiani E, Rosini F, Dotti MT, Bianchi S, Federico A, Rufa A (2013). Visual system involvement in CADASIL. J Stroke Cerebrovasc Dis.

[43] Singhal S, Bevan S, Barrick T, Rich P, Markus HS (2004). The influence of genetic and cardiovascular risk factors on the CADASIL phenotype. Brain.

[44] Ciolli L, Pescini F, Salvadori E, Del Bene A, Pracucci G, Poggesi A (2014). Influence of vascular risk factors and neuropsychological profile on functional performances in CADASIL: results from the MIcrovascular LEukoencephalopathy Study (MILES). Eur J Neurol..

[45] Hervè D, Godin O, Dufouil C, Viswanathan A, Jouvent E, Pachaï C (2009). Three-dimensional MRI analysis of individual volume of lacunes in CADASIL. Stroke.

[46] Chabriat H, Hervé D, Duering M, Godin O, Jouvent E, Opherk C (2016). Predictors of clinical worsening in cerebral autosomal dominant arteriopathy with subcortical infarcts and leukoencephalopathy: prospective cohort study. Stroke.

[47] Opherk C, Peters N, Holtmannspötter M, Gschwendtner A, Müller-Myhsok B, Dichgans M (2006). Heritability of MRI lesions volume in CADASIL: evidence for genetic modifiers. Stroke.

[48] Opherk C, Gonik M, Duering M, Malik R, Jouvent E, Hervé D (2014). Genome-wide genotyping demonstrates a polygenic risk score associated with white matter hyperintensity volume in CADASIL. Stroke.

[49] Gesierich B, Opherk C, Rosand J, Gonik M, Malik R, Jouvent E (2016). APOE ɛ2 is associated with white matter hyperintensity volume in CADASIL. J Cereb Blood Flow Met.

[50] Gunda B, Hervé D, Godin O, Bruno M, Reyes S, Alili N (2012). Effects of gender on the phenotype of CADASIL. Stroke.

[51] Pescini F, Donnini I, Cesari F, Nannucci S, Valenti R, Rinnoci V, et al. Circulating biomarkers in cerebral autosomal dominant arteriopathy with subcortical infarcts and leukoencephalopathy patients. J Stroke Cerebrovasc Dis. 2016. doi:10.1016/j.jstrokecerebrovasdis.2016.10.027. Ahead of print. 10.1016/j.jstrokecerebrovasdis.2016.10.027 27876311

[52] Lesnik Oberstein SA, Jukema JW, Van Duinen SG, Macfarlane PW, van Houwelingen HC, Breuning MH, Ferrari MD (2003). Myocardial infarction in cerebral autosomal dominant arteriopathy with subcortical infarcts and leukoencephalopathy (CADASIL). Medicine (Baltimore).

[53] Cumurciuc R, Henry P, Gobron C, Vicaut E, Bousser MG, Chabriat H (2006). Electrocardiogram in cerebral autosomal dominant arteriopathy with subcortical infarcts and leukoencephalopathy patients without any clinical evidence of coronary artery disease: a case-control study. Stroke.

[54] Rufa A, Guideri F, Acampa M, Cevenini G, Bianchi S, De Stefano N (2007). Cardiac autonomic nervous system and risk of arrhythmias in cerebral autosomal dominant arteriopathy with subcortical infarcts and leukoencephalopathy (CADASIL). Stroke.

[55] Piccirillo G, Magrì D, Mitra M, Rufa A, Zicari E, Stromillo ML (2008). Increased QT variability in cerebral autosomal dominant artieriopathy with subcortical infarcts and leukoencephalopathy. Eur J Neurol.

[56] Rufa A, Dotti MT, Franchi M, Stromillo ML, Cevenini G, Bianchi S (2005). Systemic blood pressure profile in cerebral autosomal dominant arteriopathy with subcortical infarcts and leukoencephalopathy. Stroke.

[57] Zicari E, Tassi R, Stromillo ML, Pellegrini M, Bianchi S, Cevenini G (2008). Right-to-left shunt in CADASIL patients: prevalence and correlation with clinical and MRI findings. Stroke.

[58] Mazzucco S, Anzola GP, Ferrarini M, Taioli F, Olivato S, Burlina AP (2008). Cerebral autosomal dominant arteriopathy with subcortical infarcts and leukoencephalopathy and right-to-left shunt: lack of evidence for an association in a prevalence study. Eur Neurol.

[59] Singhal S, Rich P, Markus HS (2005). The spatial distribution of MR imaging abnormalities in cerebral autosomal dominant arteriopathy with subcortical infarcts and leukoencephalopathy and their relationship to age and clinical features. AJNR Am J Neuroradiol.

[60] Auer DP, Pütz B, Gössl C, Elbel G, Gasser T, Dichgans M (2001). Differential lesion patterns in CADASIL and sporadic subcortical arteriosclerotic encephalopathy: MR imaging study with statistical parametrical group comparison. Radiology.

[61] O'Sullivan M, Jarosz JM, Martin RJ, Deasy N, Powell JF, Markus HS. MRI hyperintensities of the temporal lobe and external capsule in patients with CADASIL. Neurology. 2001;56(5):628–34. 10.1212/wnl.56.5.628 11245715

[62] Lee YC, Liu CS, Chang MH, Lin KP, Fuh JL, Lu YC, et al. Population-specific spectrum of NOTCH3 mutations, MRI features and founder effect of CADASIL in Chinese. J Neurol. 2009;256(2):249–55. 10.1007/s00415-009-0091-3 19242647

[63] Duering M, Csanadi E, Gesierich B, Jouvent E, Hervé D, Seiler S (2013). Incident lacunes preferentially localize to the edge of white matter hyperintensities: insight into the pathophysiology of cerebral small vessel disease. Brain.

[64] Lesnik Oberstein SA, van den Boom R, van Buchem MA, van Houwelingen HC, Bakker E, Vollebregt E (2001). Cerebral microbleeds in CADASIL. Neurology.

[65] Dichgans M, Holtmannspötter M, Herzog J, Peters N, Bergmann M, Yousry TA (2002). Cerebral microbleeds in CADASIL: a gradient-echo magnetic resonance imaging and autopsy study. Stroke.

[66] Rinnoci V, Nannucci S, Valenti R, Donnini I, Bianchi S, Pescini F (2013). Cerebral hemorrhages in CADASIL: Report of four cases and a brief review. J Neurol Sci..

[67] Choi JC, Song SK, Lee JS, Kang SY, Kang JH. Diversity of stroke presentation in CADASIL: study from patients harboring the predominant NOTCH3 mutation R544C. J Stroke Cerebrovasc Dis. 2013;22(2):126–31. 10.1016/j.jstrokecerebrovasdis.2011.07.002 21852154

[68] Stromillo ML, Dotti MT, Battaglini M, Mortilla M, Bianchi S, Plewnia K (2009). Structural and metabolic brain abnormalities in preclinical cerebral autosomal dominant arteriopathy with subcortical infarcts and leucoencephalopathy. J Neurol Neurosurg Psychiat.

[69] Viswanathan A, Godin O, Jouvent E, O’Sullivan M, Gschwendtner A, Peters N (2010). Impact of MRI markers in subcortical vascular dementia: a multi-modal analysis in CADASIL. Neurobiol Aging.

[70] Duering M, Zieren N, Hervé D, Jouvent E, Reyes S, Peters N (2011). Strategic role of frontal white matter tracts in vascular cognitive impairment: a voxel-based lesion-symptom mapping study in CADASIL. Brain.

[71] De Guio F, Mangin JF, Duering M, Ropele S, Chabriat H, Jouvent E (2015). White matter edema at the early stage of cerebral autosomal-dominant arteriopathy with subcortical infarcts and leukoencephalopathy. Stroke.

[72] Cognat E, Cleophax S, Domenga-Denier V, Joutel A (2014). Early white matter changes in CADASIL: evidence of segmental intramyelinic oedema in a pre-clinical mouse model. Acta Neuropathol Commun..

[73] Rossi Espagnet MC, Romano A, Carducci F, Calabria LF, Fiorillo M, Orzi F (2012). Grey matter volume alterations in CADASIL: a voxel-based morphometry study. J Headache Pain.

[74] Lambert C, Sam Narean J, Benjamin P, Zeestraten E, Barrick TR, Markus HS (2015). Characterising the grey matter correlates of leukoaraiosis in cerebral small vessel disease. Neuroimage Clin..

[75] Mascalchi M, Pantoni L, Giannelli M, Valenti R, Bianchi A, Pracucci G (2016). Diffusion tensor imaging to map brain microstructural changes in CADASIL. J Neuroimaging.

[76] Baykara E, Gesierich B, Adam R, Tuladhar AM, Biesbroek JM, Koek HL (2016). A novel imaging marker for small vessel disease based on skeletonization of white matter tracts and diffusion histograms. Ann Neurol.

[77] O’Sullivan M, Barrick TR, Morris RG, Clark CA, Markus HS (2005). Damage within a network of white matter regions underlies executive dysfunction in CADASIL. Neurology.

[78] Molko N, Pappata S, Mangin JF, Poupon F, LeBihan D, Bousser MG (2002). Monitoring disease progression in CADASIL with diffusion magnetic resonance imaging: a study with whole brain histogram analysis. Stroke.

[79] Holtmannspötter M, Peters N, Opherk C, Martin D, Herzog J, Brückmann H (2005). Diffusion magnetic resonance histograms as a surrogate marker and predictor of disease progression in CADASIL: a two-year follow-up study. Stroke.

[80] Gunda B, Porcher R, Duering M, Guichard JP, Mawet J, Jouvent E (2014). ADC histograms from routine DWI for longitudinal studies in cerebral small vessel disease: a field study in CADASIL. PLoS One.

[81] Ruchoux MM, Chabriat H, Bousser MG, Baudrimont M, Tournier-Lasserve E (1994). Presence of ultrastructural arterial lesions in muscle and skin vessels of patients with CADASIL. Stroke.

[82] Markus HS, Martin RJ, Simpson MA, Dong YB, Ali N, Crosby AH (2002). Diagnostic strategies in CADASIL. Neurology.

[83] Malandrini A, Gaudiano C, Gambelli S, Berti G, Serni G, Bianchi S (2007). Diagnostic value of ultrastructural skin biopsy studies in CADASIL. Neurology.

[84] Morroni M, Marzioni D, Ragno M, Di Bella P, Cartechini E, Pianese L (2013). Role of electron microscopy in the diagnosis of CADASIL syndrome: a study of 32 patients. PLoS One.

[85] Joutel A, Andreux F, Gaulis S, Domenga V, Cecillon M, Battail N (2000). The ectodomain of the Notch3 receptor accumulates within the cerebrovasculature of CADASIL patients. J Clin Invest.

[86] Ishiko A, Shimizu A, Nagata E, Takahashi K, Tabira T, Suzuki N (2006). Notch3 ectodomain is a major component of granular osmiophilic material (GOM) in CADASIL. Acta Neuropathol.

[87] Tikka S, Mykkänen K, Ruchoux MM, Bergholm R, Junna M, Pöyhönen M (2009). Congruence between NOTCH3 mutations and GOM in 131 CADASIL patients. Brain.

[88] Arboleda-Velasquez JF, Manent J, Lee JH, Tikka S, Ospina C, Vanderburg CR (2011). Hypomorphic Notch 3 alleles link Notch signaling to ischemic cerebral small-vessel disease. Proc Natl Acad Sci U S A.

[89] Lesnik Oberstein SA, van Duinen SG, van den Boom R, Maat-Schieman ML, van Buchem MA, van Houwelingen HC (2003). Evaluation of diagnostic NOTCH3 immunostaining in CADASIL. Acta Neuropathol.

[90] Joutel A, Favrole P, Labauge P, Chabriat H, Lescoat C, Andreux F (2001). Skin biopsy immunostaining with a Notch3 monoclonal antibody for CADASIL diagnosis. Lancet.

[91] Craggs LJ, Yamamoto Y, Deramecourt V, Kalaria RN (2014). Microvascular pathology and morphometrics of sporadic and hereditary small vessel diseases of the brain. Brain Pathol.

[92] Craggs LJ, Yamamoto Y, Ihara M, Fenwick R, Burke M, Oakley AE (2014). White matter pathology and disconnection in the frontal lobe in cerebral autosomal dominant arteriopathy with subcortical infarcts and leukoencephalopathy (CADASIL). Neuropathol Appl Neurobiol.

[93] Joutel A, Corpechot C, Ducros A, Vahedi K, Chabriat H, Mouton P (1997). Notch3 mutations in cerebral autosomal dominant arteriopathy with subcortical infarcts and leukoencephalopathy (CADASIL), a Mendelian condition causing stroke and vascular dementia. Ann N Y Acad Sci..

[94] Escary JL, Cécillon M, Maciazek J, Lathrop M, Tournier-Lasserve E, Joutel A (2000). Evaluation of DHPLC analysis in mutational scanning of Notch3, a gene with high G-C content. Hum Mutat.

[95] Larsson C, Lardelli M, White, Lendhal U (1994). The human NOTCH1,2, and 3 genes are located at chromosome position 9q34, 1012-p11, and 19p13.2-p13.1 in regions of neoplasia-associated translocation. Genomics.

[96] Joutel A, Vahedi K, Corpechot C, Troesch A, Chabriat H, Vayssière C (1997). Strong clustering and stereotyped nature of Notch3 mutations in CADASIL patients. Lancet..

[97] Federico A, Bianchi S, Dotti MT (2005). The spectrum of mutations for CADASIL diagnosis. Neurol Sci.

[98] Dotti MT, Federico A, Mazzei R, Bianchi S, Scali O, Conforti FL (2005). The spectrum of Notch3 mutations in 28 Italian CADASIL families. J Neurol Neurosurg Psychiatry.

[99] Rutten JW, Boon EM, Liem MK, Dauwerse JG, Pont MJ, Vollebregt E (2013). Hypomorphic NOTCH3 alleles do not cause CADASIL in humans. Hum Mutat.

[100] Finsterer J (2007). Neuromuscular implications in CADASIL. Cerebrovasc Dis.

[101] Tuominen S, Juvonen V, Amberla K, Jolma T, Rinne JO, Tuisku S (2001). Phenotype of a homozygous CADASIL patient in comparison to 9 age-matched heterozygous patients with the same R133C Notch3 mutation. Stroke.

[102] Liem MK, Lesnik Oberstein SA, Vollebregt MJ, Middelkoop HA, van der Grond J, Helderman-van den Enden AT (2008). Homozygosity for a NOTCH3 mutation in a 65-year-old CADASIL patient with mild symptoms: a family report. J Neurol.

[103] Soong BW, Liao YC, Tu PH, Tsai PC, Lee IH, Chung CP (2013). A homozygous NOTCH3 mutation p.R544C and a heterozygous TREX1 variant p.C99MfsX3 in a family with hereditary small vessel disease of the brain. J Chin Med Assoc.

[104] Vinciguerra C, Rufa A, Bianchi S, Sperduto A, De Santis M, Malandrini A (2014). Homozygosity and severity of phenotypic presentation in a CADASIL family. Neurol Sci.

[105] Abou Al-Shaar H, Qadi N, Al-Hamed MH, Meyer BF, Bohlega S (2016). Phenotypic comparison of individuals with homozygous or heterozygous mutation of NOTCH3 in a large CADASIL family. J Neurol Sci..

[106] Rutten JW, Haan J, Terwindt GM, van Duinen SG, Boon EM, Lesnik Oberstein SA (2014). Interpretation of NOTCH3 mutations in the diagnosis of CADASIL. Expert Rev Mol Diagn..

[107] Bianchi S, Dotti MT, Gallus GN, D’Eramo C, Di Donato I, Bernardi L (2013). First deep intronic mutation in the NOTCH3 gene in a family with late-onset CADASIL. Neurobiol Aging.

[108] Nakamura K, Ago T, Tsuchimoto A, Noda N, Nakamura A, Ninomiya T (2015). A CADASIL-like case with a novel non cysteine mutation of the NOTCH3 gene and granular deposits in the renal arterioles. Case Rep Neurol Med..

[109] Ueda A, Ueda M, Nagatoshi A, Hirano T, Ito T, Arai N (2015). Genotypic and phenotypic spectrum of CADASIL in Japan: the experience at a referral center in Kumamoto University from 1997 to 2014. J Neurol.

[110] Wollenweber FA, Hanecker P, Bayer-Karpinska A, Malik R, Bäzner H, Moreton F (2015). Cysteine-sparing CADASIL mutations in NOTCH3 show proaggregatory properties in vitro. Stroke.

[111] Moccia M, Mosca L, Erro R, Cervasio M, Allocca R, Vitale C (2015). Hypomorphic NOTCH3 mutation in a family with CADASIL features. Neurobiol Aging.

[112] Pippucci T, Maresca A, Magini P, Cenacchi G, Donadio V, Palombo F (2015). Homozygous NOTCH3 null mutation and impaired NOTCH3 signaling in recessive early-onset arteriopathy and cavitating leukoencephalopathy. EMBO Mol Med.

[113] Fouillade C, Chabriat H, Riant F, Mine M, Arnoud M, Magy L (2008). Activating NOTCH3 mutation in a patient with small-vessel-disease of the brain. Hum Mutat.

[114] Martignetti JA, Tian L, Li D, Ramirez MC, Camacho-Vanegas O, Camacho SC (2013). Mutations in PDGFRB cause autosomal-dominant infantile myofibromatosis. Am J Med Genet.

[115] Gripp KW, Robbins KM, Sobreira NL, Witmer PD, Bird LM, Avela K (2015). Truncating Mutations in the last exon of NOTCH3 cause lateral meningocele syndrome. Am J Med Genet Part A.

[116] Reyes S, Kurtz A, Hervé D, Tournier-Lasserve E, Chabriat H (2012). Presymptomatic genetic testing in CADASIL. J Neurol.

[117] Goldman JS (2015). Genetic testing and counseling in the diagnosis and management of young-onset dementias. Psychiatr Clin North Am.

[118] Milunsky A, Konialis C, Shim SH, Maher TA, Spengos K, Ito M (2005). The prenatal diagnosis of cerebral autosomal dominant arteriopathy with subcortical infarcts and leukoencephalopathy (CADASIL) by mutation analysis. Prenat Diagn.

[119] Konialis C, Hagnefelt B, Kokkali G, Pantos C, Pangalos C (2007). Pregnancy following preimplantation genetic diagnosis of cerebral autosomal dominant arteriopathy with subcortical infarcts and leukoencephalopathy (CADASIL). Prenat Diagn.

[120] Rutten JW, Dauwerse HG, Peters DJ, Goldfarb A, Venselaar H, Haffner C (2016). Therapeutic NOTCH3 cysteine correction in CADASIL using exon skipping: in vitro proof of concept. Brain.

[121] Keverne JS, Low WC, Ziabreva I, Court JA, Oakley AE, Kalaria RN (2007). Cholinergic neuronal deficits in CADASIL. Stroke.

[122] Shobha N, Fang J, Hill MD (2013). Do lacunar strokes benefit from thrombolysis? Evidence from the Registry of the Canadian Stroke Network. Int J Stroke.

[123] Dichgans M, Markus HS, Salloway S, Verkkoniemi A, Moline M, Wang Q (2008). Donepezil in patients with subcortical vascular cognitive impairment: a randomised double-blind trial in CADASIL. Lancet Neurol.

[124] Joshi S, Yau W, Kermode A (2017). CADASIL mimicking multiple sclerosis: The importance of clinical and MRI red flags. J Clin Neurosci..

[125] Carone DA. CADASIL and multiple sclerosis: A case report of prolonged misdiagnosis. Appl Neuropsychol Adult. 2016;11:1–4. 10.1080/23279095.2016.1214132 27712110

[126] Yanagawa S, Ito N, Arima K, Ikeda S (2002). Cerebral autosomal recessive arteriopathy with subcortical infarcts and leukoencephalopathy. Neurology.

[127] Perneczky R, Tene O, Attems J, Giannakopoulos P, Ikram MA, Federico A (2016). Is the time ripe for new diagnostic criteria of cognitive impairment due to cerebrovascular disease? Consensus report of the International Congress on Vascular Dementia working group. BMC Med..

[128] Plastino M, Messina D, Cristiano D, Lombardo G, Bosco D (2015). Pathological gambling associated with CADASIL: an unusual manifestation. Neurol Sci.

[129] Haddad N, Ikard C, Hiatt K, Shanmugam V, Schmidley J (2015). Recurrent status epilepticus as the primary neurological manifestation of CADASIL: a case report. Epilepsy Behav Case Rep..

[130] Lågas PA, Juvonen V (2001). Schizophrenia in a patient with cerebral autosomally dominant arteriopathy with subcortical infarcts and leucoencephalopathy (CADASIL disease). Nord J Psychiatry.

[131] Ho CS, Mondry A (2015). CADASIL presenting as schizophreniform organic psychosis. Gen Hosp Psychiatry.

[132] Sicurelli F, Dotti MT, De Stefano N, Malandrini A, Mondelli M, Bianchi S (2005). Peripheral neuropathy in CADASIL. J Neurol.

[133] Kang SY, Oh JH, Kang JH, Choi JC, Lee JS (2009). Nerve conduction studies in cerebral autosomal dominant arteriopathy with subcortical infarcts and leukoencephalopathy. J Neurol.

[134] Lackovic V, Bajcetic M, Lackovic M, Novakovic I, Labudović Borović M, Pavlovic A (2012). Skin and sural nerve biopsies: ultrastructural findings in the first genetically confirmed cases of CADASIL in Serbia. Ultrastruct Pathol.

[135] de la Peña P, Bornstein B, del Hoyo P, Fernández-Moreno MA, Martín MA, Campos Y (2001). Mitochondrial dysfunction associated with a mutation in the Notch3 gene in aCADASIL family. Neurology.

[136] Malandrini A, Albani F, Palmeri S, Fattapposta F, Gambelli S, Berti G (2002). Asymptomatic cores and paracrystalline mitochondrial inclusions in CADASIL. Neurology.

[137] Dotti MT, De Stefano N, Bianchi S, Malandrini A, Battisti C, Cardaioli E (2004). A novel NOTCH3 frameshift deletion and mitochondrial abnormalities in a patient with CADASIL. Arch Neurol.

[138] Viitanen M, Sundström E, Baumann M, Poyhonen M, Tikka S, Behbahani H (2013). Experimental studies of mitochondrial function in CADASIL vascular smooth muscle cells. Exp Cell Res.

[139] Ragno M, Pianese L, Morroni M, Cacchiò G, Manca A, Di Marzio F (2013). “CADASIL coma” in an Italian homozygous CADASIL patient: comparison with clinical and MRI findings in age-matched heterozygous patients with the same G528C NOTCH3 mutation. Neurol Sci..

[140] Hartley J, Westmacott R, Decker J, Shroff M, Yoon G (2010). Childhood-onset CADASIL: clinical, imaging, and neurocognitive features. J Child Neurol.

[141] Cleves C, Friedman NR, Rothner AD, Hussain MS (2010). Genetically confirmed CADASIL in a pediatric patient. Pediatrics.

[142] Kumar SK, Mahr G (1997). CADASIL presenting as bipolar disorder. Psychosomatics.

[143] Collongues N, Derache N, Blanc F, Labauge P, de Seze J, Defer G (2012). Inflammatory-like presentation of CADASIL: a diagnostic challenge. BMC Neurol..

[144] Rufa A, Cerase A, Monti L, Battisti C, Forte F, Federico A (2008). Acute vestibular syndrome in a patient with cerebral autosomal dominant leukoencephalopathy with subcortical infarcts and leukoencephalopathy (CADASIL). J Neurol Sci.

[145] Bentley P, Wang T, Malik O, Nicholas R, Ban M, Sawcer S (2011). CADASIL with cord involvement associated with a novel and atypical NOTCH3 mutation. J Neurol Neurosurg Psychiat.

[146] Pinto WB, Souza PV, Oliveira A (2015). Longitudinally extensive transverse myelopathy in a patient with CADASIL. Arq Neuropsiquiatr.

[147] Sathe S, DePeralta E, Pastores G, Kolodny EH (2009). Acute confusional migraine may be a presenting feature of CADASIL. Headache.

[148] Monteiro C, Barros J, Taipa R, Pereira-Monteiro J (2012). Sporadic hemiplegic migraine with normal imaging as the initial manifestation of CADASIL. Cephalalgia.

[149] Pantoni L, Pescini F, Inzitari D, Dotti MT (2005). Postpartum psychiatric disturbances as an unrecognized onset of CADASIL. Acta Psychiatric Scand.

[150] Ragno M, Berbellini A, Cacchiò G, Manca A, Di Marzio F, Pianese L (2013). Parkinsonism is a late, not rare, feature of CADASIL: a study on Italian patients carrying the R1006C mutation. Stroke.

[151] Pradotto L, Orsi L, Mencarelli M, Caglio M, Lauro D, Milesi A (2016). Recurrent transient global amnesia as presenting symptoms of CADASIL. Clin Case Rep.

[152] Weller M, Dichgans J, Klockgether T (1998). Acetazolamide-responsive migraine in CADASIL. Neurology..

[153] Forteza AM, Brozman B, Rabinstein AA, Romano JG, Bradley WG (2001). Acetazolamide for the treatment of migraine with aura in CADASIL. Neurology..

[154] Donnini I, Nannucci S, Valenti R, Pescini F, Bianchi S, Inzitari D (2012). Acetazolamide for the prophylaxis of migraine in CADASIL: a preliminary experience. J Headache Pain.

[155] Martikainen MH, Roine S (2012). Rapid improvement of a complex migrainous episode with sodium valproate in a patient with CADASIL. J Headache Pain..

[156] Chabriat H, Pappata S, Ostergaard L, Clark CA, Pachot-Clouard M, Vahedi K (2000). Cerebral hemodynamics in CADASIL before and after acetazolamide challenge assessed with MRI bolus tracking. Stroke..

[157] Huang L, Yang Q, Zhang L, Chen X, Huang Q, Wang H (2010). Acetazolamide improves cerebral hemodynamics in CADASIL. J Neurol Sci..

[158] Park SA, Yang CY, Choi SS, Kim WH (2011). Assessment of cerebral hemodynamics to acetazolamide using brain perfusion SPECT in cerebral autosomal dominant arteriopathy with subcortical infarcts and leukoencephalopathy. Clin Nucl Med..

[159] Fujiwara Y, Mizuno T, Okuyama C, Nagakane Y, Watanabe-Hosomi A, Kondo M (2012). Simultaneous impairment of intracranial and peripheral artery vasoreactivity in CADASIL patients. Cerebrovasc Dis..

[160] Peters N, Freilinger T, Opherk C, Pfefferkorn T, Dichgans M (2007). Effects of short term atorvastatin treatment on cerebral hemodynamics in CADASIL. J Neurol Sci..

[161] Peters N, Freilinger T, Opherk C, Pfefferkorn T, Dichgans M (2008). Enhanced L-arginine-induced vasoreactivity suggests endothelial dysfunction in CADASIL. J Neurol..

[162] De Maria R, Campolo J, Frontali M, Taroni F, Federico A, Inzitari D (2014). Effects of sapropterin on endothelium-dependent vasodilation in patients with CADASIL: a randomized controlled trial. Stroke..

[163] Liu XY, Gonzalez-Toledo ME, Fagan A, Duan WM, Liu Y, Zhang S (2015). Stem cell factor and granulocyte colony-stimulating factor exhibit therapeutic effects in a mouse model of CADASIL. Neurobiol Dis..

